# Wild/Woodland Mushroom Poisoning: The Experience of Bucharest Emergency Hospital-Retrospective Study of ER 2023–2024 Presentations

**DOI:** 10.3390/jof11080578

**Published:** 2025-08-03

**Authors:** Bogdan Oprita, Mihai Ciprian Neacsu, Bogdan Alexandru Dinu, Ionut Olaru, Ruxandra Oprita

**Affiliations:** 1 Emergency Department, Clinical Emergency Hospital of Bucharest, 105402 Bucharest, Romania; 2 Faculty of Medicine, University of Medicine and Pharmacy “Carol Davila”, 050474 Bucharest, Romania; 3 Romania Gastroenterology Department, Clinical Emergency Hospital of Bucharest, 105402 Bucharest, Romania

**Keywords:** mushrooms, intoxication, encephalopathy, cytolysis, muscarinic, gastrointestinal disorders, hepato-renal disorders

## Abstract

The global trend of increasing mushroom consumption, combined with traditional practices in Romania and other Eastern European countries of collecting and consuming “wild mushrooms”, may contribute to the rising incidence of emergency presentations due to inedible mushroom poisoning. This study aims to identify the clinical features of mushroom poisoning by retrospectively analyzing 47 cases presented to the Emergency Department of the Bucharest Emergency Hospital between 2023 and 2024. The methodology consists of a retrospective cohort study including all patients presented to the Emergency Department of the Bucharest Emergency Hospital with symptoms following mushroom ingestion between 2023 and 2024 totaling 47 cases. Conclusions: In this cohort, most cases of wild/forest mushroom poisoning (76.59%) were diagnosed during autumn, particularly in September and October. The distribution of cases was uniform with respect to both gender and urban versus rural residence. A significant proportion of patients (74.46%) required hospitalization for surveillance and/or specific treatment. The predominant clinical presentation consisted of gastrointestinal symptoms, observed in 97.87% of cases.

## 1. Introduction

Mushrooms have been an important source of nutrition since prehistoric times, as evidenced by archeological findings. Early human societies relying on hunting, fishing, and foraging developed empirical knowledge of edible fungi, often by observing animal behavior. The toxicity of certain mushroom species has also been recognized for thousands of years—one notable example being the suspected mushroom poisoning that caused the death of the Roman Emperor Claudius [1].

An article published in *The Guardian* [2] reports that mushroom sales in the United States have increased by 20% over the past decade. Similarly, according to United Nations data, global mushroom sales have tripled in the last 15 years. In line with this trend, the Clinical Emergency Hospital in Bucharest, one of the few medical centers in Romania with a dedicated toxicology department, has observed a rise in emergency presentations involving acute mushroom poisoning.

Consequently, we conducted a two-year retrospective study (2023–2024) analyzing presentations to the Emergency Department of the Bucharest Emergency Hospital, with symptoms following the ingestion of wild mushrooms. The data revealed a more than fourfold increase in reported cases, from 9 in 2023 to 38 in 2024, although no direct causality could be established.

**Figure 1:**
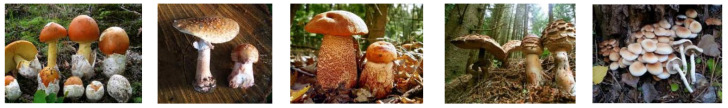
Types of edible fungi commonly identified in Romania, in order presented from left to right: “*Amanita caesarea*”, “*Amanita rubescens*”, “*Leccinum aurantiacum*”, “*Macrolepiota procera*”, and “*Agrocybe cylindracea*”.

In Romania, and in Eastern European countries collecting forest and field mushrooms during the peak season (September–October) by non-professionals for personal consumption is a long-standing tradition [3] particularly prevalent in rural areas. The most commonly encountered edible forest species in Romanian woodlands include *Amanita caesarea*, *Amanita rubescens*, *Leccinum aurantiacum*, *Macrolepiota procera,* and *Agrocybe cylindracea* [4]. These species are illustrated in Figure 1.

Insufficient knowledge regarding edible mushroom species often results in poisoning, which can be life-threatening and constitutes a significant public health concern [5]. Moreover, most toxins found in inedible mushrooms are resistant to standard thermal preparation methods [6].

The primary objective of this study is to describe the clinical and epidemiological features of mushroom poisoning based on a retrospective case series presented to the Emergency Department of the Bucharest Emergency Clinical Hospital between 2023 and 2024. Specifically, we aimed to examine the factors associated with the increasing number of cases, the demographic and clinical characteristics of affected patients, and potential indicators of severe outcomes.

Although the study is primarily descriptive and exploratory, we hypothesized that (1) older age and (2) delayed presentation to the Emergency Department (more than 24 h after ingestion) might be associated with more severe clinical courses. These hypotheses were formulated based on prior evidence suggesting that these factors may influence prognosis in mushroom poisoning cases.

The lack of statistical data on mushroom poisoning cases, in Romania and other Eastern European countries, combined with the increasing incidence of presentations in the Emergency Department constituted the main rationale for this study [7]. The growing number of emergency department visits due to mushroom poisoning in Romania highlights an underexplored public health issue, particularly in the absence of centralized toxicological surveillance or standardized treatment guidelines. While amateur foraging remains a widespread cultural practice, especially in rural and peri-urban areas, medical literature on mushroom-related intoxications in Eastern Europe remains scarce. Moreover, there is a notable lack of clinical data correlating symptom patterns with prognosis, and little is known about the demographic profile or risk factors associated with severe outcomes. This study aims to address these gaps by providing real-world data from one of the few toxicology-capable centers in the country, offering a descriptive analysis of mushroom poisoning presentations and associated clinical variables [8,9].

## 2. Materials and Methods

We conducted a retrospective cohort study by reviewing patient records from both the Emergency Department and the Toxicology Ward at the Bucharest Emergency Clinical Hospital. Patients were included if they were adults (≥18 years) who presented with acute gastrointestinal and/or neurological symptoms occurring within 48 h after self-reported ingestion of wild mushrooms between 1 January 2023 and 31 December 2024. Diagnosis was based on clinical evaluation, without toxicological confirmation, and relied on a combination of patient history, symptom pattern, and timing of symptom onset.

Patients were excluded if their symptoms could be attributed to other plausible causes, such as alcohol intoxication, foodborne illness, or drug ingestion, or if the history of mushroom ingestion was unclear or inconsistent.

Only adult patients (≥18 years), consistent with the hospital’s admission policy, were included. A total of 47 cases met the inclusion criteria, of whom 35 were hospitalized in the Toxicology Ward.

A structured database was developed to extract key clinical variables: month and year of presentation, sex, age, diagnosis, hospital admission decision, area of residence (urban vs. rural), time from ingestion to ED arrival, symptom profile (gastrointestinal, neurological, muscarinic, hepato-renal), laboratory parameters (pH, bicarbonate, AST, ALT, urea, creatinine), and duration of hospital stay. The analysis focused on the following research questions:

1. What factors contributed to the increase in mushroom poisoning cases observed in 2024?
2. Are there specific demographic or clinical variables—such as age or time to presentation—associated with poor prognosis?
3. What is the distribution of cases by gender and residence (urban vs. rural)?
4. What types of symptoms predominated at presentation, and how frequently did severe outcomes occur?
5. Were there any reported characteristics of the mushrooms ingested that could offer insight into the toxicological profile? Although the exact species could not be identified, most patients reported ingesting wild mushrooms harvested in forested or peri-urban areas, with a characteristic profile of early-onset gastrointestinal symptoms. These clinical features suggested the likely involvement of gastrointestinal irritant toxins such as muscarine or other non-lethal compounds found in *Chlorophyllum* or *Boletus* species, as discussed in the literature.

After the completion of the database, a statistical analysis was used to extract the results, which enabled the formulation of conclusions based on the study objectives. Descriptive statistical analysis was conducted to summarize the clinical and epidemiological characteristics of the included cases. Categorical variables (such as sex, symptom type, or residence area) were expressed as absolute numbers and percentages. Continuous variables (including age, hospitalization duration, and laboratory parameters) were reported as means and standard deviations. No formal hypothesis testing was applied, as the primary objective was descriptive. However, for exploratory purposes, comparisons between subgroups (e.g., severe vs. non-severe cases) were performed using the Mann–Whitney U test for continuous variables and the chi-square (χ^2^) test for categorical variables [10]. Missing data were minimal and were not subject to imputation; analysis was based on available case records [11]. Although it was not possible to identify the exact mushroom species consumed, all patients reported ingesting freshly picked, non-commercial mushrooms collected personally.

## 3. Results

In 2023 and 2024, a total of 47 patients presented to the Emergency Department of the Bucharest Clinical Emergency Hospital, with self-reported clinical symptoms following mushroom consumption. Of these, 9 cases (19.14%) were recorded in 2023 and 38 (80.86%) in 2024. This notable increase in cases provided additional support for conducting the present study. While the marked increase in 2024 is noteworthy and prompted this study, no causal interpretation should be made from this difference, as external factors such as climate, public behavior, or healthcare access were not formally assessed. The sex distribution was nearly equal with 23 females (48.93%) and 24 males (51.07%). Regarding patient residence, 29 (61.70%) lived in urban areas and 18 (38.30%) in rural areas.

**Chart 1:**
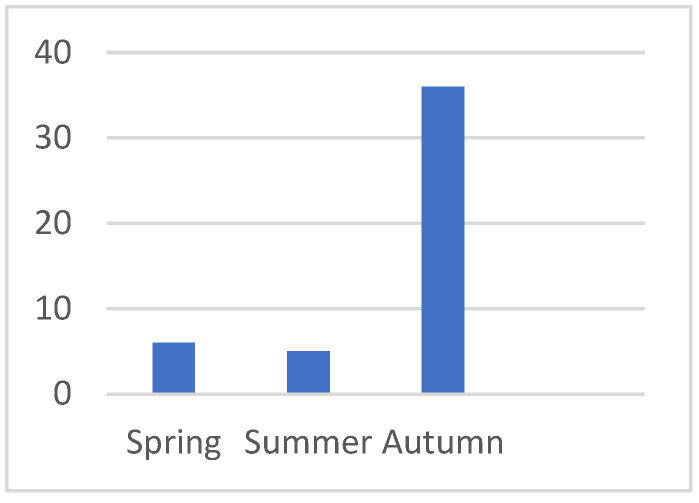
Seasonal ratio of presentation.

The seasonal distribution showed that 36 cases (76.59%) occurred in autumn (September–October), while 5 (10.63%) were recorded in summer and 6 (12.76%) in spring. *This distribution is illustrated in* Chart 1. In 43 cases (91.48%), the time from mushroom ingestion to hospital presentation was less than 24 h, while in four cases (8.52%) it exceeded 24 h. Symptoms at presentation were categorized into gastrointestinal (diarrhea, vomiting, nausea, abdominal pain), neurological (altered consciousness, myoclonus, vertigo, seizures), muscarinic (bronchospasm, bronchorrhea, bradycardia, hypersalivation), and hepato-renal (jaundice, urinary hyperchromia) manifestations. Of the 47 patients, gastrointestinal symptoms were the most common, occurring in 46 cases (97.87%), followed by neurological disorders in 14 patients (29.78%). Then, muscarinic disorders were present in two patients with bronchospasm (4.25%) and one patient had jaundice, classified as a hepato-renal sign and symptom (2.12%). The distribution of symptoms among patients is summarized in Table 1. Clinical picture at presentation.

Of the 47 patients, 35 (74.46%) were admitted to the Toxicology Ward, while 12 (25.54%) were discharged under the supervision of their family physician. For inpatients, the average length of hospitalization was 1.87 days. Three of the hospitalized patients required advanced intensive care measures (oro-tracheal intubation and mechanical ventilation) and were classified as severe cases, with an average hospital stay of 7 days. In all of these three cases defined as serious, the duration of presentation to the Emergency Reception Unit from the time of ingestion was more than 24 h (out of the total of the four cases presented >24 h from ingestion). The mean age for these severe cases was 64.66 years, notably higher than the cohort’s overall average of 47.85 years. Patients hospitalized for less than two days had a mean age of 42.92 years, compared to 61.14 years for those admitted longer than two days. Although these patterns suggest that delayed presentation and older age may be associated with more severe clinical outcomes, the small number of severe cases limits the ability to draw firm conclusions. These observations should be interpreted with caution and may serve as the basis for future hypothesis-driven studies. The final aspect analyzed involved laboratory investigations, focusing on the following parameters: pH, HCO_3_, liver transaminases (AST, ALT), urea, and creatinine. The values were recorded both at the time of presentation during T1, and the highest value of the parameters during the stay in the Toxicology ward (for inpatients), defined as T2 time. No deviations from physiological limits were observed in pH or bicarbonate levels in any of the patients. Regarding the parameters expressing renal function, urea and creatinine, changes above physiological values were identified in only one patient (classified as a severe case in the analyzed cohort), with elevated values recorded both during T1 and T2. In the case of liver transaminases, values above the physiological limits of AST and ALT were identified in three of the patients (all three representing patients categorized as severe case) during T2 and in only two patients (of the three identified) at the time of presentation in the ED during T1. Examples of these abnormal hepato-renal values are summarized in Table 2.

The Bucharest Emergency Clinical Hospital provides care exclusively to adult patients (over 18 years old). In all cases the mushrooms consumed were self-harvested and not commercially purchased.

## 4. Discussion

According to published studies, wild/forest mushroom ingestion remains a diagnostically non-specific cause of poisoning in Europe, often based on self-reported symptoms [12]. The correlation between mushroom ingestion and the varied clinical presentation often complicates diagnosis. In Europe, the number of reported cases varies significantly across studies, influenced by cultural practices and by the geographic and climatic distribution of inedible mushroom species [13]. Although over 5000 species of fungi have been identified worldwide, only 25–30% are fully described and less than 3% are considered toxic [14]. This study found that 76.59% of patients developed symptoms after ingesting wild mushrooms in September and October, indicating a strong seasonal pattern during autumn. These findings suggest that in Romania, wild mushroom poisonings occur predominantly in autumn, although globally, the fruiting season of wild mushrooms extends from March to November. No statistically significant differences were observed in gender or place of residence (urban vs. rural), indicating a uniform distribution among the analyzed population, suggesting a uniform distribution within the population, especially considering the urban location of the Bucharest Emergency Hospital.

The marked increase in cases observed in 2024 may reflect multiple converging factors. A favorable climate for mushroom growth during early autumn, combined with economic constraints that push individuals to forage for food, could explain the rise in forest mushroom consumption and subsequent poisonings. Anecdotal evidence and previous studies from Eastern Europe suggest that socioeconomic factors and limited access to toxicological education play an important role in the persistence of foraging-related poisoning [15]. Additionally, the growing popularity of nature-based leisure activities in peri-urban populations may lead to greater exposure to unidentified wild mushrooms.

Although no specific mushroom species were identified in this cohort, the high prevalence of gastrointestinal symptoms with short latency (less than 6–8 h in most cases) suggests the involvement of *Agaricus* spp., *Chlorophyllum molybdites*, or *Boletus satanas*, all of which are known to cause acute gastroenteritis [16,17]. While toxicological confirmation or morphological identification of mushroom species was not performed in this study, the symptom profile provides indirect clues regarding the likely toxins involved. The predominance of gastrointestinal symptoms with rapid onset (within 6–8 h) in nearly all patients suggests the involvement of low-molecular-weight gastrointestinal irritants, such as those found in species of *Chlorophyllum molybdites*, *Boletus satanas*, or *Agaricus xanthodermus* [1,16]. These agents are known to cause self-limited gastroenteritis and are frequently implicated in similar seasonal outbreaks in Central and Eastern Europe [12]. While these correlations remain hypothetical, they offer a plausible toxicological framework in the absence of species-level identification. This clinical pattern is consistent with previous case series from Central and Eastern Europe [18,19], where seasonal peaks in autumn were similarly observed. However, in the absence of toxicological confirmation, these remain hypothetical correlations. Comparative data from other European countries support the seasonal and clinical trends observed in our study. For instance, a retrospective analysis from Serbia reported a similar autumnal peak in wild mushroom poisoning cases, with 81% of patients presenting between September and October, and gastrointestinal symptoms dominating the clinical picture in over 90% of cases [19]. A Swiss study covering 11 years likewise found that most cases occurred during early autumn, with Chlorophyllum molybdites and Agaricus spp. frequently suspected in non-fatal gastrointestinal presentations [12]. In Southern Germany, an increase in mycetism incidence was also observed between 2015 and 2022, with elderly patients being overrepresented among those requiring hospitalization or intensive care [13]. Italy, one of the countries with robust poison center reporting systems, has documented consistent annual peaks during late summer and autumn, with a predominance of Amanita phalloides-related hepatotoxic syndromes, contrasting with the gastroenteritis-dominated profiles in Eastern Europe [7]. These geographic variations may reflect differences in foraging practices, species distribution, and public health awareness. Placing our findings in this broader context highlights both the shared epidemiological features across European regions and the importance of local environmental and social factors that shape poisoning patterns. Examples of the demographic distribution by sex and living environment are presented in Table 3.

Gastrointestinal symptoms (nausea, vomiting, diarrhea, and abdominal pain) were the most frequently observed, affecting 97.87% of patients presenting with wild mushroom poisoning. Most patients presenting to the ED with symptoms following wild mushroom ingestion required hospitalization (74.46% of patients). The average hospital stay among inpatients was 1.87 days. Notably, 24 of the 35 admitted patients had hospitalizations shorter than this average, indicating a skewed distribution influenced by a small number of severe cases. In these cases, hospitalization served primarily symptomatic treatment and follow-up of laboratory parameters to detect potential hepato-renal complications. Three cases were identified as severe, based on the requirement for advanced intensive care measures in the Toxicology ward including oro-tracheal intubation and mechanical ventilation. The average age of these cases was 64.66 years compared to 47.85 years in the overall cohort. However, due to the small sample size, no statistically significant association could be established between advanced age and poor prognosis. All three severe cases presented to the Emergency Department more than 24 h after ingestion, in contrast to the rest of the cohort, where 91.48% of patients presented within the first 24 h, compared to the presentation within the first 24 h after ingestion applicable to 91.48% of the analyzed cohort. Although delayed presentation (>24 h) could be a potential negative prognostic factor, this hypothesis could not be confirmed due to the limited number of severe cases. This study has several limitations: the fungal species involved could not be identified; data were restricted to adult patients, due to the hospital’s profile; and the number of severe cases was insufficient for statistical validation. First, the retrospective design inherently limited data consistency and detail, as documentation relied entirely on medical records not initially intended for research purposes. Important clinical elements such as precise timing of symptom onset, amount of mushrooms consumed, or co-ingestions could not be verified or quantified. Second, the small number of severe cases prevented the identification of statistically significant predictors of poor outcomes. As a result, hypotheses regarding the impact of delayed presentation or advanced age could not be fully validated and should be interpreted cautiously. Third, the inability to identify the exact mushroom species consumed represents a major limitation, especially in distinguishing between toxic syndromes with similar gastrointestinal onset but different prognoses and treatment approaches [15]. Moreover, the inability to identify the ingested mushroom species introduces a significant degree of clinical uncertainty. Without species-level identification, it becomes difficult to correlate symptom patterns with known toxic syndromes, which may vary substantially in terms of latency, severity, and required management. This limitation complicates both the interpretation of presenting symptoms and the prediction of clinical outcomes. In particular, the distinction between benign gastrointestinal irritants and potentially hepatotoxic species such as *Amanita phalloides* remains speculative in the absence of toxicological confirmation or mycological expertise. Consequently, clinical decision-making must often rely solely on temporal and syndromic patterns, which may not always reflect the true toxic potential of the ingested fungi. This issue is common in real-world emergency settings, where mycological expertise or toxicological confirmation is rarely available, particularly when patients present with nonspecific symptoms and no remaining mushroom samples for analysis. This reflects a broader public health challenge in many Eastern European countries, where amateur foraging remains common and mycological expertise is rarely available in emergency settings [16]. Another important limitation is the absence of toxicological confirmation. Although this reflects real-world clinical practice in emergency settings, it may affect diagnostic accuracy and complicate the identification of specific toxidromes. Furthermore, due to retrospective design, the exact time interval between ingestion and Emergency Department presentation could not be determined for all patients. Future studies should statistically assess whether delayed presentation (>24 h) and advanced patient age constitute negative prognostic factors, using a larger, statistically powered cohort.

## 5. Conclusions

Poisoning from wild mushrooms remains a public health concern in Europe, largely due to the difficulty in correlating non-specific clinical symptoms with mushroom ingestion and the lack of accurate species identification.

In Romania, most cases occurred during autumn, particularly in September and October, reflecting the seasonal pattern associated with wild mushroom harvesting.

Gastrointestinal symptoms—nausea, vomiting, abdominal pain, and diarrhea—were predominant, affecting nearly all patients, confirming their role as the primary clinical manifestations in mushroom poisonings.

Although most patients required hospitalization, the average length of stay was under two days, suggesting a generally favorable clinical course requiring only symptomatic treatment and observation.

Severe cases were rare but appeared to be associated with delayed hospital presentation (>24 h post-ingestion) and older patient age. These factors may represent negative prognostic indicators, although statistical significance could not be demonstrated due to the limited sample size. These findings also underscore the need for public health measures aimed at prevention, particularly in regions where amateur mushroom foraging is culturally widespread. Educational campaigns targeting at-risk populations, along with improved toxicology training for emergency department staff, may help reduce the incidence and severity of mushroom-related poisonings.

## Figures and Tables

**Table 1:** Clinical picture at presentation.

Signs and Symptoms at the Time of Presentation in the ED			
Disorder	Definition	Number of Patients	Percentage
GASTROINTESTINAL	Diarrhea, vomiting, nausea, abdominal pain	46	97.87%
NEUROLOGICAL	Altered consciousness, myoclonus, vertigo, convulsions	14	29.78%
MUSCARINIC	Bronchospasm, bronchorrhea, bradycardia, hypersalivation	2	4.25%
HEPATO-RENAL	Jaundice, urinary hyperchromia	1	2.12%

**Table 2:** Examples of pathological values of the hepato-renal system; Abbreviations and units: T1—values at presentation in the Emergency Department; T2—peak values during hospitalization; AST—Aspartate aminotransferase (U/L); ALT—Alanine aminotransferase (U/L); Urea (mg/dL); Creatinine (mg/dL).

Patient	T1		T2		T1		T2	
	Urea	Creatinine	Urea	Creatinine	AST	ALT	AST	ALT
**M/70 YEARS**	203	2.25	177	1.97	360	1299	367	1170
**F/42 YEARS**	81	1.03	35	0.6	2084	4304	711	2797
**M82 YEARS**	53	0,9	28	0.94	27.4	15.9	81.74	75.59

**Table 3:** Distribution of cases by sex and living environment (urban/rural).

	Number of Patients	Percentage of Patients
**Sex of patient**	23 women/24 men	48.93% women/51.07% men
**Urban/rural residence**	29 urban/18 rural	61.70% urban/38.30% rural

## Data Availability

The original data presented in the study are included in the article, further inquiries can be directed to the corresponding author.
